# Application of the CDK9 inhibitor FIT-039 for the treatment of KSHV-associated malignancy

**DOI:** 10.1186/s12885-023-10540-y

**Published:** 2023-01-20

**Authors:** Tetsunori Sakamoto, Masahiko Ajiro, Akira Watanabe, Shingo Matsushima, Keiji Ueda, Masatoshi Hagiwara

**Affiliations:** 1 Department of Anatomy and Developmental Biology, Kyoto University Graduate School of Medicine, Building C, 3Rd Floor, Yoshida-Konoe Cho, Sakyo-Ku, Kyoto, 606-8501 Japan; 2 Present address: Japanese Red Cross Otsu Hospital, Otsu, 520-8511 Japan; 3 Department of Drug Discovery Medicine, Kyoto University Graduate School of Medicine, Kyoto, 606-8501 Japan; 4 Medical Innovation Center, Kyoto University Graduate School of Medicine, Kyoto, 606-8397 Japan; 5 Division of Virology, Osaka University Graduate School of Medicine, Suita, 565-0871 Japan

**Keywords:** Kaposi’s sarcoma-associated herpesvirus, Primary effusion lymphoma, FIT-039, Cyclin-dependent kinase 9, BCBL-1 xenograft

## Abstract

Chronic infection with Kaposi’s sarcoma-associated herpes virus (KSHV) in B lymphocytes causes primary effusion lymphoma (PEL), the most aggressive form of KSHV-related cancer, which is resistant to conventional chemotherapy. In this study, we report that the BCBL-1 KSHV^+^ PEL cell line does not harbor oncogenic mutations responsible for its aggressive malignancy. Assuming that KSHV viral oncogenes play crucial roles in PEL proliferation, we examined the effect of cyclin-dependent kinase 9 (CDK9) inhibitor FIT-039 on KSHV viral gene expression and KSHV^+^ PEL proliferation. We found that FIT-039 treatment impaired the proliferation of KSHV^+^ PEL cells and the expression of KSHV viral genes in vitro. The effects of FIT-039 treatment on PEL cells were further evaluated in the PEL xenograft model that retains a more physiological environment for the growth of PEL growth and KSHV propagation, and we confirmed that FIT-039 administration drastically inhibited PEL growth in vivo. Our current study indicates that FIT-039 is a potential new anticancer drug targeting KSHV for PEL patients.

**Supplementary Information:**

The online version contains supplementary material available at 10.1186/s12885-023-10540-y.

## Introduction

Kaposi’s sarcoma-associated herpesvirus (KSHV) is an oncogenic virus, and its chronic infection is associated with the development and progression of primary effusion lymphoma (PEL), Kaposi’s sarcoma (KS), and multicentric Castleman’s disease (MCD) [1–3]. Among them, PEL is the most aggressive form of KSHV-related malignancy, with a typical median survival period of less than six months [4–8]. KSHV-associated PEL is a diffuse large B cell lymphoma caused by a chronic KSHV infection in B lymphocytes. Combination chemotherapies, such as CHOP (cyclophosphamide, doxorubicin, vincristine, and prednisone) and EPOCH (etoposide, prednisone, vincristine, cyclophosphamide, and doxorubicin), in addition to an antiretroviral therapy when patients are positive for human immunodeficiency virus-1 (HIV-1), are often used as treatment. However, in most cases, PEL is resistant to these regimens, and effective therapeutic options for patients with PEL are currently limited.

The KSHV genome encodes several viral oncogenes required for transformation of host cells [1, 2, 9], and the expression of KSHV lytic and latent genes is suggested to be important for progression [7]. However, genomic information on KSHV-associated cancers remain largely uncharacterized; therefore, the contribution of KSHV viral oncogenes to tumor progression and the appropriate molecular target against KSHV-related cancer remain unclear. Contributions of somatic mutations and viral factors for PEL malignancy are currently not fully understood, and a detailed characterization of PEL cells may provide a clue to identify a new strategy for better therapeutics. Thus, in this study we examined the somatic mutations in the KSHV^+^ PEL cell lines by whole genome sequencing.

## Materials and methods

### Cell lines and culture

KSHV^+^ human PEL cell lines, BCBL-1 (obtained from NIH AIDS Research & Reference Reagent Program, Rockville, MD, #3233) and BC-3 (obtained from American Type Culture Collection (ATCC), Manassas, VA, CRL-2277), Raji Burkitt lymphoma cells (obtained from Japanese Collection of Research Bioresources Cell Bank (JCRB), Osaka, Japan, #JCRB9012), and B95-8 monkey lymphocytes (obtained from JCRB, #JCRB9123) were maintained in RPMI 1640 (Nacalai Tesque, Kyoto, Japan) supplemented with 10% fetal bovine serum (FBS), penicillin (100 U/mL), and streptomycin (100 μg/mL). Human keratinocytes HaCaT cells (established by Dr. Norbert E. Fusenig [10] and obtained from Dr. Peter M. Howley of Harvard Medical School) were maintained in DMEM (Nacalai Tesque, Kyoto, Japan) supplemented with 10% FBS serum, penicillin (100 U/mL), and streptomycin (100 μg/mL). All cells were maintained in a humidified incubator at 37 °C and 5% CO_2_. Lytic reactivation was performed using 20 ng/mL 12-O-tetradecanoylphorbol-13-acetate (TPA), and 1.5 mM sodium butyrate for BC-3 cells, and 0.6 mM sodium valproate for BCBL-1 cells as reported previously [11, 12]. The integrity of the cell lines used in this study was confirmed using short tandem repeat analysis (Takara Bio Inc., Shiga, Japan), and the cells were confirmed to be negative for mycoplasma by PCR.

### Cell counting

The relative cell number in the cell culture was determined with the WST-8 assay using Cell Count Reagent SF (Nacalai Tesque). Cell counting for ascites cells from BCBL-1 xenografts were counted using a hemocytometer.

### Compound

The FIT-039 used in this study was synthesized by IWAKI SEIYAKU Co., Ltd., Tokyo, Japan.

### Western blot

Cells were collected in a conical tube and lysed in Laemmli buffer, followed by sonication and heat denaturation. Protein lysates were separated by SDS-PAGE and transferred to polyvinylidene fluoride membranes. Protein-bound membranes were then incubated with blocking buffer containing 5% skim milk in Tris-buffered saline, followed by incubation with following antibodies: anti-RTA mouse monoclonal antibody α50A [13], anti-ORF57 mouse monoclonal antibody (kindly provided by Drs. Vladimir Majerciak and Zhi-Ming Zheng of National Cancer Institute, MD) [14–16], anti-c-Myc rabbit monoclonal antibody (ab32072, Abcam, Cambridge, UK), anti-Bcl-2 rabbit monoclonal antibody (Abcam), anti-Bcl-XL rabbit monoclonal antibody (Abcam), or anti-β-actin (ACTB) mouse monoclonal antibody (AC-15) (Santa Cruz Biotechnology, Inc. Dallas, TX). Membranes were subsequently incubated with anti-mouse IgG goat polyclonal antibody with HRP (Abcam) or anti-rabbit IgG goat polyclonal antibody with HRP (GE Health care, Chicago, Il). Chemiluminescent detection was conducted using a Super Signal West Pico and Femto (Thermo Fisher Scientific, Waltham, MA, USA) and a ChemiDoc imaging system (Bio-Rad, Hercules, CA, USA). Uncropped blot images are shown in Supplementary Fig. S1.

### RT-PCR

Total RNA was extracted using Sepasol reagent (Nacalai Tesque) with DNase treatment, and used as a template for reverse transcription using PrimeScript Reverse Transcriptase (Takara Bio Inc.) and random hexamer (Takara Bio Inc.). The reverse transcription products were subjected to PCR using the primer sets listed in Supplementary Table S1. PCR was performed using ExTaq DNA polymerase (Takara Bio Inc.). PCR products were applied to agarose gel electrophoresis and detected with ethidium bromide staining. Uncropped gel images are shown in Supplementary Fig. S1.

### Immunocytostaining

BCBL-1 cells were fixed with 4% paraformaldehyde (Nacalai Tesque), permeabilized with 0.2% Triton X-100 in Dulbecco’s phosphate-buffered saline (D-PBS) and blocked with 3% bovine serum albumin (BSA). The cells were then incubated with anti-RTA mouse monoclonal antibody diluted in D-PBS containing 3% BSA. After washing with D-PBS, the cells were incubated with AlexaFluor488-conjugated anti-mouse IgG 1:500 in 3% BSA. The nuclear DNA was stained with Hoechst 33,342 (Thermo Fisher Scientific). The stained cells were analyzed using a BZ-X710 fluorescence microscope and BZ-X analyzer software (Keyence, Osaka, Japan).

### Immunohistostaining

Tissue sections were prepared from paraffin blocks of 10% neutral formalin-fixed peritoneum, liver, and spleen tissues from mice. Following deparaffinization and antigen retrieval, tissue slide samples were blocked with 3% BSA in PBS, and incubated with anti-GAPDH rabbit polyclonal antibody (ab128915, abcam), which binds to human GAPDH but not to that of mouse. Following wash with PBS-T, AlexaFluor488-conjugated anti-rabbit IgG were incubated, and further washed with PBS-T. Tissue sections were then counterstained for nuclear DNA with Hoechst 33,342 (Thermo Fisher Scientific), and sealed with cover glass. 

### Xenograft study

Immune-compromised NOD/SCID mice were purchased from CLEA Japan, Inc. (Tokyo, Japan). Since PEL patients are dominantly male, we examined the xenograft test using male mice. Six-week-old male NOD/SCID mice were intraperitoneally (*i.p*.) inoculated with 1 $\times$ 10^7^ BCBL-1 cells suspended in 200 µL PBS, and on the same day, the mice were subsequently initiated for *i.p*. administration with 75, 150, or 300 mg/kg body weight (mg/kg-BW) FIT-039 in 0.005% methyl cellulose (MC)/PBS or solvent only (vehicle) thrice a week (*t.i.w.*). Mice were sacrificed on day 43, and ascites volume, ascites cell number, ascites cell frequency, and spleen length were analyzed. An age-adjusted control of NOD/SCID mice without BCBL-1 inoculation or administration of FIT-039 or vehicle were also analyzed. An additional set of 6-week-old male NOD/SCID mice was used for BCBL-1 inoculation and *i.p.* administration of FIT-039 (300 mg/kg-BW) or vehicle control. Upon sacrifice, mice were euthanized using carbon dioxide following AVMA guideline on euthanasia, and the KSHV load was analyzed in ascites and plasma. The animal study was approved by the Kyoto University Graduate School of Medicine, and performed in accordance with ARRIVE guidelines.

### Flow cytometry (FCM)

Ascites cells, collected from mice, were applied to 70 µm cell strainer, and suspended in 10 mL PBS. For those with a small volume of ascites or no apparent ascites, 5 mL of PBS was added to the intraperitoneal cavity and washed with PBS solution. After centrifugation (500 g$\times$, 5 min), cells were resuspended in ammonium-chloride-pottasium (ACK) buffer (Themo Fisher Scientific) and incubated for 5 min at RT. Cells were then centrifuged (500 g$\times$, 5 min) and resuspended in FCM buffer (PBS with 0.5% BSA and 5 mM EDTA). Cells were applied to 70 µm cell strainer, and 1 $\times$ 10^6^ of the resulting cells were applied to mouse and human Fc blockers (2.4G2 for mouse and Fc1.3216 for human, BD Biosciences, Franklin Lakes, NJ) and incubated with anti-human CD45-APC (HI30, BioLegend, San Diego, CA) and anti-human CD38-PE (HIT2, BioLegend), or IgG1κ-APC (MOPC-21, BioLegend) and IgG1κ-PE (M075-5, Medical & Biological Laboratories Co., Ltd., Tokyo, Japan) as isotype controls, for 30 min at 4 °C. Cells were washed thrice with 500 µL FCM buffer, resuspended in 500 µL FCM buffer, and subjected to FCM using BD Accuri C6 (BD Biosciences). FCM data were analyzed with FlowJo v.10.8.0 (BD Biosciences).

### qPCR for copy number determination

Lytically induced BCBL-1 cells were incubated with or without FIT-039. After 96 h of incubation, the culture supernatant was centrifuged and applied to a 0.45 μm filter. Viral DNA was purified using a ZR Viral DNA Kit (Zymo Research, Irvine, CA, USA). For the xenograft mouse study, total DNA in the ascites and plasma of the xenograft model mice was collected using a DNeasy Blood & Tissue Kit (QIAGEN, Hilden, Germany). The KSHV *RTA* copy number was quantified using TaqMan RT-PCR with a copy number standard for reference to the Ct values. The primers and probe used for the real-time PCR are listed in Supplementary Table S1.

### Whole genome sequencing

Whole genome sequencing of BCBL-1 and BC-3 cells was performed using NovaSeq6000 (Illumina, Inc. San Diego, CA). Library construction was performed using a TruSeq DNA PCR-free library (Illumina, Inc.). Single nucleotide polymorphisms were subtracted by referring to variants with a minor allele frequency of ≥ 1% in variant information from Tohoku Medical Megabank Organization, HGVD [17], and ExAC [18]. The pathogenicity of a given mutation was determined using JAX CKB (https://ckb.jax.org/), CIViC (https://civicdb.org/home), COSMIC (https://cancer.sanger.ac.uk/cosmic), and ClinVar (https://www.ncbi.nlm.nih.gov/clinvar/). As a result, we found 755 and 916 variants, respectively for BCBL-1 and BC-3, as those included in the COSMIC Gene Census. Subsequent manual curation revealed 2 mutants each for BCBL-1 and BC-3 were annotated as potential driver mutations.

### Statistical analysis

Numerical data are shown as means ± standard error (SE) or box and whisker plot with 5–95 percentile, as indicated in each graph. Student’s t-test or one-way ANOVA with Turkey’s multiple comparisons test, calculated with GraphPad Prism 7.05 (GraphPad Software, San Diego, CA), was applied for statistical evaluations and is indicated in individual figure legends. Statistical significance was set at *p* < 0.05.

## Result

### Profiling of somatic mutations of BCBL-1 and BC-3 cells suggests lack of driver mutation to explain growth promotion

To investigate whether the KSHV^+^ PEL cell line, BCBL-1 and BC-3, harbor driver mutations responsible for its aggressive malignancy, we conducted whole-genome sequencing. We identified two mutations in oncogenes for each cell line: E501K of B-Raf proto-oncogene serine/threonine kinase (BRAF) and S303X of checkpoint kinase 2 (CHEK2) for BCBL-1 cells, and R108K of epidermal growth factor receptor (EGFR) and Q217X of RB transcriptional corepressor 1 (RB1) for BC-3 cells (Table 1). Although BC-3 cells harbored two known driver mutations, R108K EGFR [19] and Q217X RB1, contributions of identified mutations to malignancy could be moderate for BCBL-1. E501K BRAF is known to be functionally equivalent to the wild-type form based on *ETS like-1* (*ELK*) transactivation activity [20], and contribution of S303X CHEK2 to cell growth is unclear [21], although it is suggested to promote transformation through impaired DNA damage response. The fact that typical driver mutation was not found in BCBL-1 genome suggests that KSHV viral factors may cooperate with other somatic mutations.

**Table 1:** Somatic mutations of oncogenes in BCBL-1 and BC-3

Cell line	Gene	Mutation	Genotype	VAF^a^
BCBL-1	*BRAF*	E501K	Heterozygous	62%
BCBL-1	*CHEK2*	S303X	Heterozygous	61%
BC-3	*EGFR*	R108K	Heterozygous	65%
BC-3	*RB1*	Q217X	Homozygous	100%

^a^Variant allele frequency (VAF): mutated copy number divided by total copy number

### Inhibition of KSHV lytic gene expression with FIT-039

**Fig. 1:**
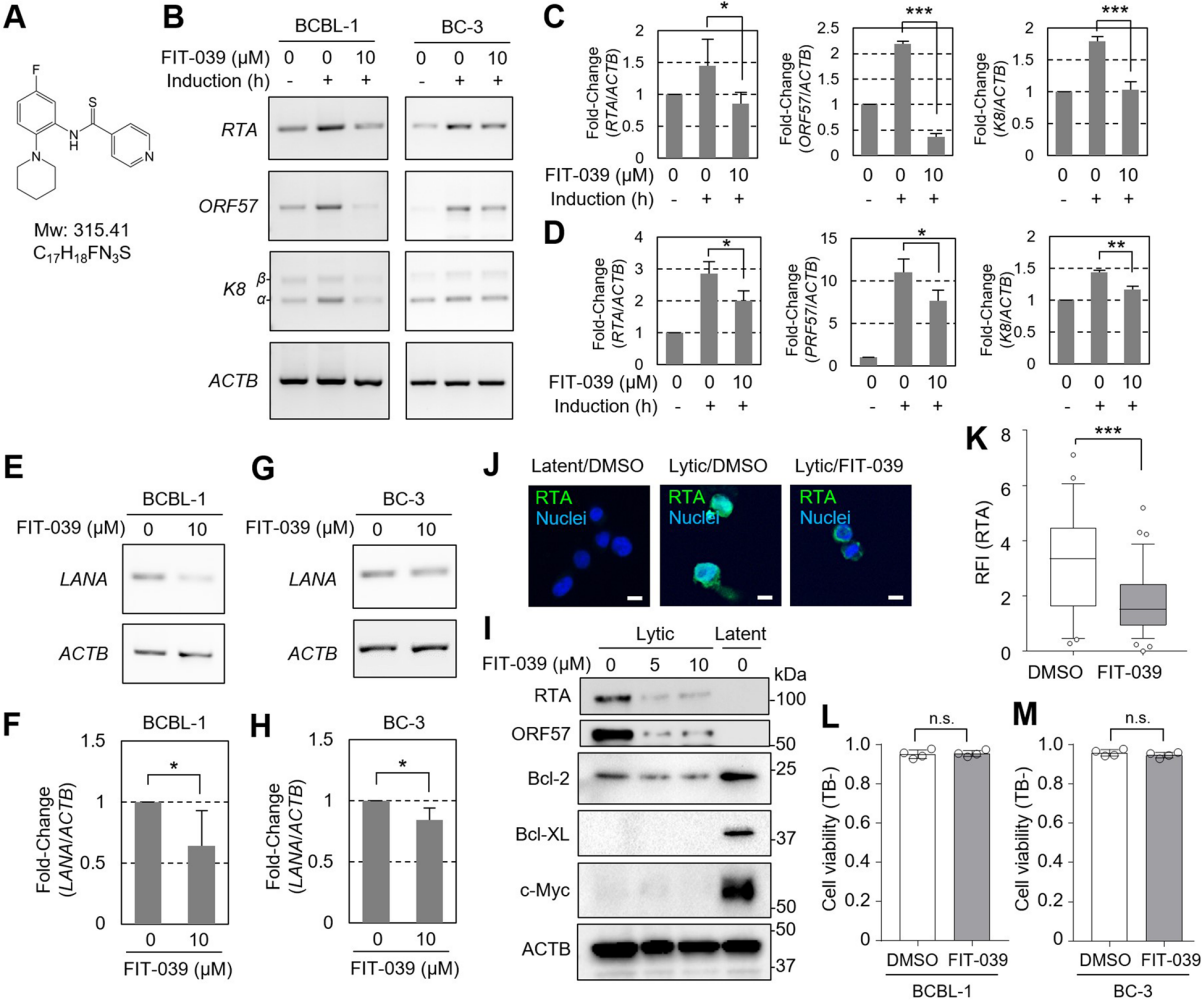
Inhibition of KSHV viral gene expression with the FIT-039. **A** Chemical structure of the FIT-039. **B-D** RT-PCR results are indicated for the KSHV viral gene expression in the PEL cells. Latent phase and lytic phase with or without 10 µM FIT-039 treatment for 48 h are analyzed for KSHV.^+^ BCBL-1 and BC-3 cells, and representative data are shown in (**B**) and quantitative data for three replicates are shown in (**C**) for BCBL-1 and in (**D**) for BC-3. Data were analyzed for three replicates and original gel images are shown in supplementary Fig. S1 and S2. *ACTB* served as a loading control. **E–H** RT-PCR results are indicated for KSHV latent gene *LANA* and *ACTB* as a loading control. Representative data and quantification were indicated for BCBL-1 (**E**, **F**), and BC-3 (**G**, **H**). Data were analyzed for three replicates and original gel images are shown in supplementary Fig. S3. **I** Western blot of lytic gene products and anti-apoptotic proteins, Bcl-2, Bcl-XL, and c-Myc, in the BC-3 cells with lytic induction for 48 h with 0, 5, and 10 µM FIT-039 treatment, as well as latent cells treated with the 0.1% DMSO for 48 h. Data from one experiment was shown. Full gel images are shown in supplementary Fig. S3. **J-K** Representative immunocytostaining images (**J**) and quantification for the relative fluorescent intensity (RFI) of RTA (**K**) in the BCBL-1 cells lytically induced for 48 h with or without the FIT-039 treatment (10 µM) for 48 h (*n* = 34 for 0 μM and *n* = 45 for 10 μM). Box and whisker plot with 5–95 percentile are indicated in (**K**). **L-M** Cell viability was determined with trypan blue test, counting trypan blue-negative cell population (TB-), for BCBL-1 (**L**) and BC-3 (**M**) with or without FIT-039 treatment at 10 µM for 96 h. Data from four replicates were plotted. *, *p* < 0.05; **, *p* < 0.01; ***, *p* < 0.001 by one-way ANOVA with Turkey’s multi-comparison test in (**C**) and (**D**). *, *p* < 0.05; ***, *p* < 0.001; n.s., *p* ≥ 0.05 by two-tailed Student’s t-test in (**F**), (**H**), (**K**), (**L**), and (**M**)

We and others have reported that transcription of viral genes is prone to depend on the host cellular transcriptional regulator, positive transcription elongation factor (P-TEFb), a complex of cyclin-dependent kinase 9 (CDK9) and cyclin T. The CDK9 inhibitor FIT-039 shows a wide spectrum of antiviral activities for herpesviruses by targeting viral transcripts [22–25]. Moreover, FIT-039 indicates safe profiles in the past pre-clinical and clinical evaluations [22–27], thereby we here chose this compound for assessment of CDK9 inhibition. Therefore, we examined the expression of the major KSHV lytic genes in BCBL-1 and BC-3 KSHV^+^ PEL cell lines following treatment with FIT-039 (Fig. 1A). We found that FIT-039 treatment suppressed the transcription of KSHV lytic genes, *RTA/ORF50*, *ORF57*, and *K8/K-bZIP*, induced upon lytic stimulation (Fig. 1B-D). Furthermore, we observed *LANA*, a major latent transcript of KSHV, was suppressed in PEL cell lines treated with the FIT-039 (Fig. 1E-H). For RTA and ORF57, suppressed protein expression was also confirmed by western blotting (Fig. 1I). In addition, FIT-039 did not affect representative anti-apoptotic proteins, including Bcl-2, Bcl-XL, and c-Myc (Fig. 1I), supporting assumption that FIT-039 primarily suppresses KSHV viral gene expressions. Moreover, suppressed RTA protein expression was also confirmed by immunocytostaining (Fig. 1J and K). On the other hand, FIT-039 treatment did not induce an acute induction of cell death (Fig. 1L, M), suggesting that FIT-039 impairs cell cycle progression rather than inducing apoptosis. Together, these data indicate that the KSHV viral gene expressions are dependent on CDK9 and susceptible to FIT-039 treatment, as observed for other viruses [22–25].

**Fig. 2:**
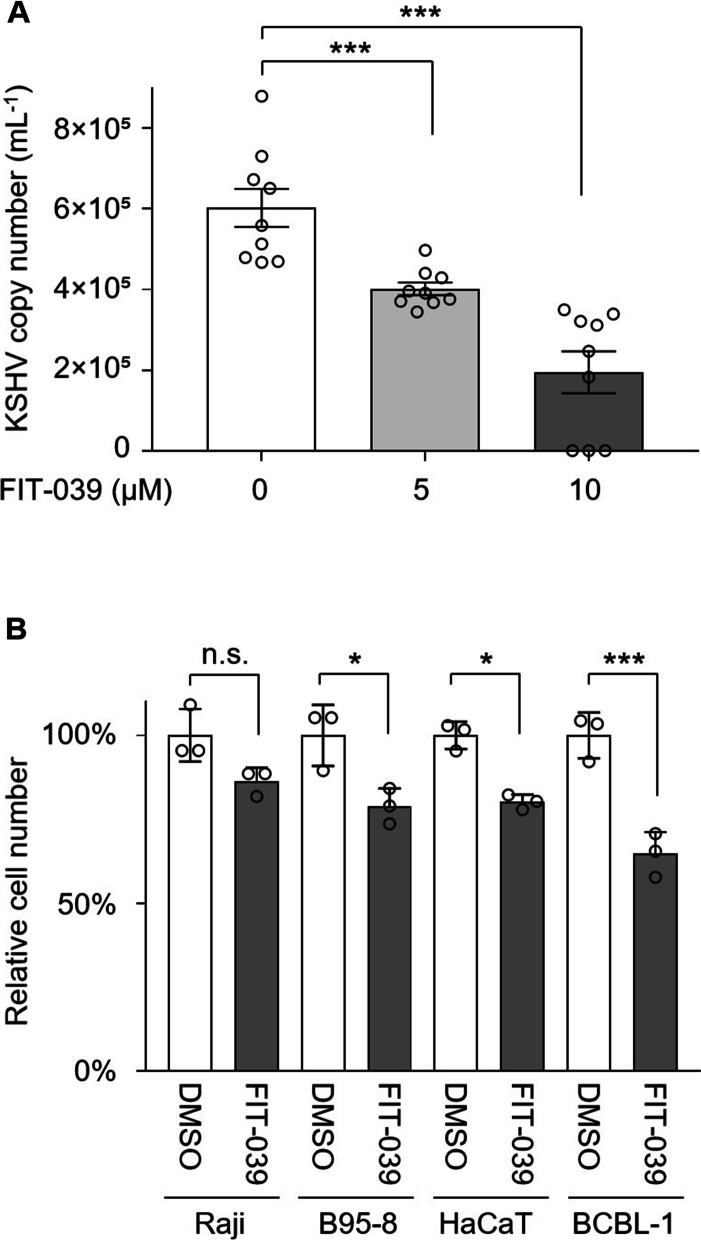
Suppression of the viral propagation in cells with the FIT-039. **A** The KSHV viral loads were determined by the TaqMan PCR for *RTA* in culture media supernatants of the BCBL-1 cells. The KSHV copy number was determined from Ct values at 96 h after lytic induction with 0, 5, or 10 μM FIT-039 treatment. Data are shown for *n* = 9 for each condition. **B** The relative cell number of BCBL-1 (PEL, KSHV^+^), Raji (Burkitt lymphoma, KSHV^−^), B95-8 (monkey lymphocytes, KSHV^−^), and HaCaT (human keratinocytes KSHV^−^), determined with the WST-8 assay at 48 h after the 0 or 10 µM FIT-039 treatment are indicated. Data from three replicates are indicated for each condition. Mean ± SE are shown in (**A**) and mean ± SD in (**B**). 0.1% DMSO served as 0 µM control in (**A**) and (**B**). *, *p* < 0.05; ***, *p* < 0.001; n.s., *p* ≥ 0.05 by one-way ANOVA with Turkey’s multiple comparisons test in (**A**) and (**B**)

We then examined whether suppression of KSHV viral gene expression by FIT-039 treatment also hinders the propagation of KSHV. We found dose-dependent reduction in KSHV viral load in culture media of BCBL-1 cells treated with FIT-039 (33% and 68% reduction at 5 and 10 µM, respectively; Fig. 2A). This is consistent with the above observation for the suppression of KSHV lytic genes (Fig. 1B-D), which are essential for the lytic replication of KSHV. We further examined the viability of BCBL-1 and KSHV^−^ cells (Raji Burkitt lymphoma cells, B95-8 primate lymphocytes, and HaCaT human keratinocytes) following FIT-039 treatment for 96 h. Although a moderate (B95-8 and HaCaT) or no significant reduction (Raji) was found in cell number was found for KSHV^−^ cells, KSHV^+^ BCBL-1 cells indicated a larger extent of cell number reduction than KSHV^−^ cells (Fig. 2B), suggesting KSHV^+^ cells are more sensitive to FIT-039 treatment for a growth suppression.

### The FIT-039 administration suppressed proliferation of KSHV^+^ PEL in xenograft model

**Fig. 3:**
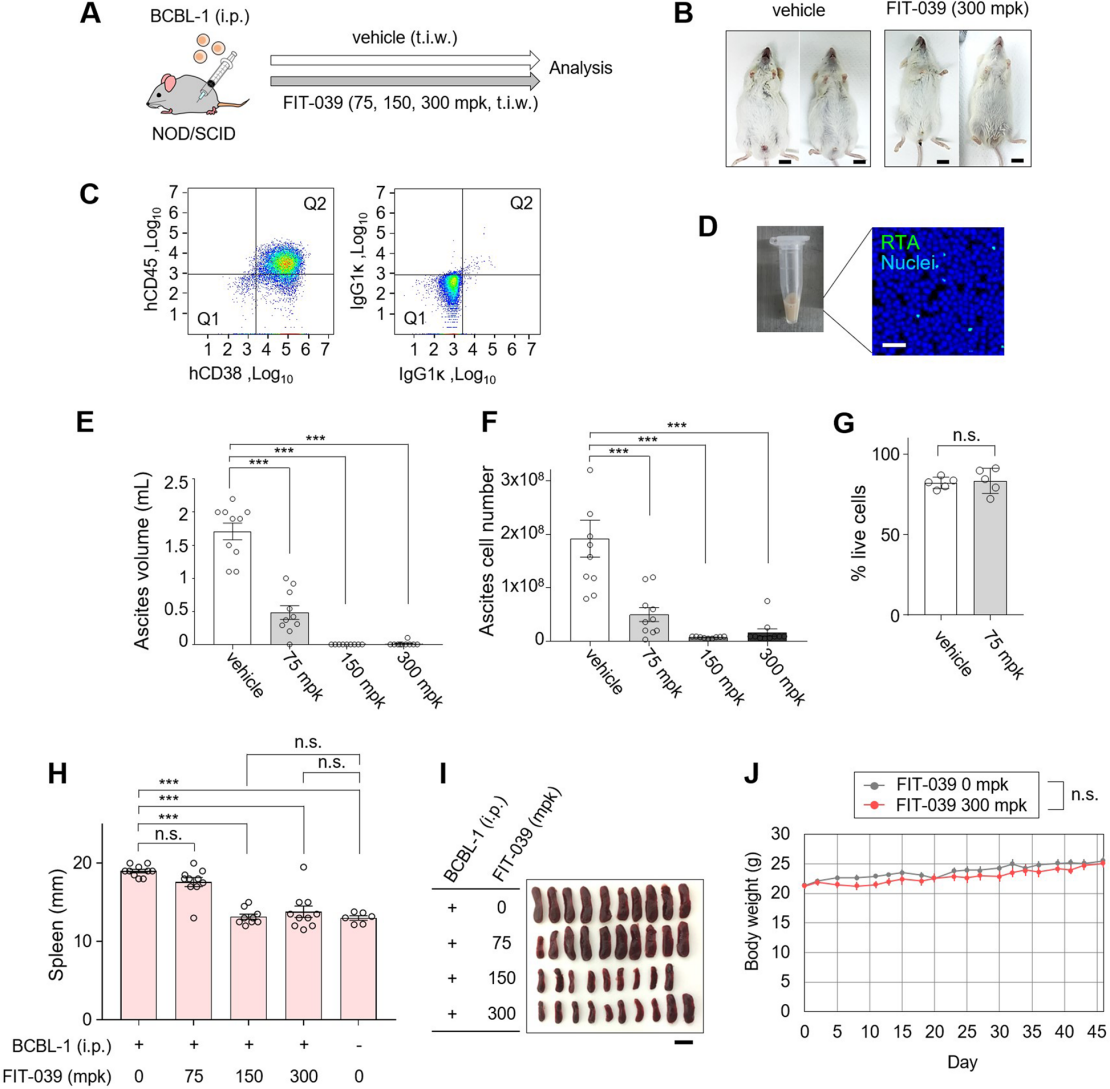
The FIT-039 administration rescued the PEL malignancy in the xenograft model. **A** The schema of the PEL xenograft study. The BCBL-1 cells were intraperitoneally (*i.p.*) inoculated in NOD/SCID mice, followed by *i.p.* administration of the vehicle or FIT-039 (75, 150, or 300 mg/kg-BW), thrice a week (*t.i.w.*). **B** Representative pictures of the mice at day 45. Bars, 1 cm. **C** FCM plot for ascites from the vehicle-treated mice at day 43. Plots for PE-human CD38 and APC-human CD45 (left) and isotype controls (right) are shown. **D** Pictures of the ascites and immunochemistry for RTA are shown. Bars, 50 µm. **E**-**F** The ascites volume (**E**) and total cell number (**F**) are plotted for PEL xenograft mice treated with the FIT-039 at 0, 75, 150, and 300 mg/kg-BW, *i.p.*, *t.i.w.* for 43 days. **G** Live cell rates of xenografted BCBL-1 in ascites (0 or 75 mg/kg-BW *i.p.* administration of FIT-039) are determined by FCM through FSC-SSC plot of human CD38 (hCD38)-positive cells. **H** Length of the spleens from the BCBL-1 xenograft mice after FIT-039 administration (0, 75, 150, or 300 mg/kg-BW) for 43 days are plotted. Spleen lengths of the age-adjusted control mice, without the xenograft or the administration of the 0.5% MC or FIT-039, are also indicated. **I** Pictures of spleens from FIT-039-administered mice were shown. Bar indicates 1 cm. **J** Body weights of NOD/SCID mice administered with 300 mg/kg-BW FIT-039 or vehicle *t.i.w.*, without the inoculation of BCBL-1 xenograft. *n* = 10, 10, 9, and 10 for 0, 75, 150, and 300 mg/kg-BW in (**E**) and (**F**), *n* = 10, 10, 9, and 10 for 0, 75, 150, and 300 mg/kg-BW, and *n* = 6 for the group without the xenograft or FIT-039 administration in (**H**), and *n* = 6 for vehicle and 300 mg/kg-BW in (**J**). mpk for mg/kg-BW in (**A**-**J**). Mean ± SE are shown; n.s., *p* ≥ 0.05; ***, *p* < 0.001 by one-way ANOVA with Turkey’s multiple comparisons test in (**E**), (**F**) and (**H**). Mean ± SE are shown; n.s., *p* ≥ 0.05 by two-tailed Student’s t-test in (**G**) and (**J**)

Lytic replication occurs naturally in KSHV-associated cancer, and lytic cells are considered to play a major role in cancer cell proliferation through paracrine factors [7, 28–32]. Therefore, the contribution of viral replication to PEL growth has not been fully evaluated in vitro, where nearly all cells are in the latent phase in the absence of any chemical stimuli. Therefore, we applied a PEL xenograft model, retaining an autonomous lytic replication cycle, for the further evaluation of FIT-039 for inhibition of PEL proliferation. Immune-deficient NOD/SCID mice were *i.p.* inoculated with BCBL-1 cells, and subsequently administered FIT-039 (75, 150, or 300 mg/kg body weight (mg/kg-BW)) or vehicle control (0 mg/kg-BW) *t.i.w.* (Fig. 3A). By day 45, we observed ascites in mice treated with the vehicle control (Fig. 3B). Ascites were chylous and filled with cells double-positive for human CD45 and CD38 in a flow cytometer [33], which were expressed on BCBL-1 cells, confirming that these cells were derived from xenografted BCBL-1 cells (Fig. 3C). Immunocytochemistry of the chylous ascites cells demonstrated that 0.3–0.6% of cells were positive for lytic marker RTA (Fig. 3D), indicating the occurrence of lytic replication.

We found that the *i.p.* administration of FIT-039 at 75 mg/kg-BW FIT-039 treatment suppressed the chylous ascites for both volume and cell number (by 71.7% and 74.0%, respectively, Fig. 3E, F), and administration at the 150 or 300 mg/kg-BW reached further suppression (by 100% or 99.4% for ascites volumes and 96.2% and 91.5% for cell numbers, respectively, Fig. 3E, F). We also found that live cell rates were not affected in ascites with FIT-039 administration (Fig. 3G), consistent with in vitro observations (Fig. 1L, 1M). In addition, we examined PEL xenograft-associated splenomegaly [33]. We found that splenomegaly was undetectable in mice treated with 150 or 300 mg/kg-BW *i.p.* FIT-039, and the spleens from the 150 and 300 mg/kg-BW FIT-039-treated mice were comparable to those from untreated healthy mice (Fig. 3H for plots of all groups, and 3I for representative pictures for mice with FIT-039 administration), ruling out the possibility of retardation in spleen tissues. Additionally, we observed a possible infiltration in peritoneum of BCBL-1-xenografted mice, and its detection rate was also lower in FIT-039-administered mice over those administered with vehicle (90% for 0 mg/kg-BW and 60% for 300 mg/kg-BW, supplementary Table S2 and supplementary Fig. S4). Moreover, in order to confirm the safety of *i.p.* administration of FIT-039, we conducted 300 mg/kg-BW *i.p.* administration in NOD/SCID mice without xenograft cells. We found that there was no significant change in body weight at each day point until day 46 (Fig. 3J), confirming that 300 mg/kg-BW FIT-039 *i.p.* administration had no apparent adverse effects. The safety of FIT-039 administration is also concordant with those of previous reports [23–25].

**Fig. 4:**
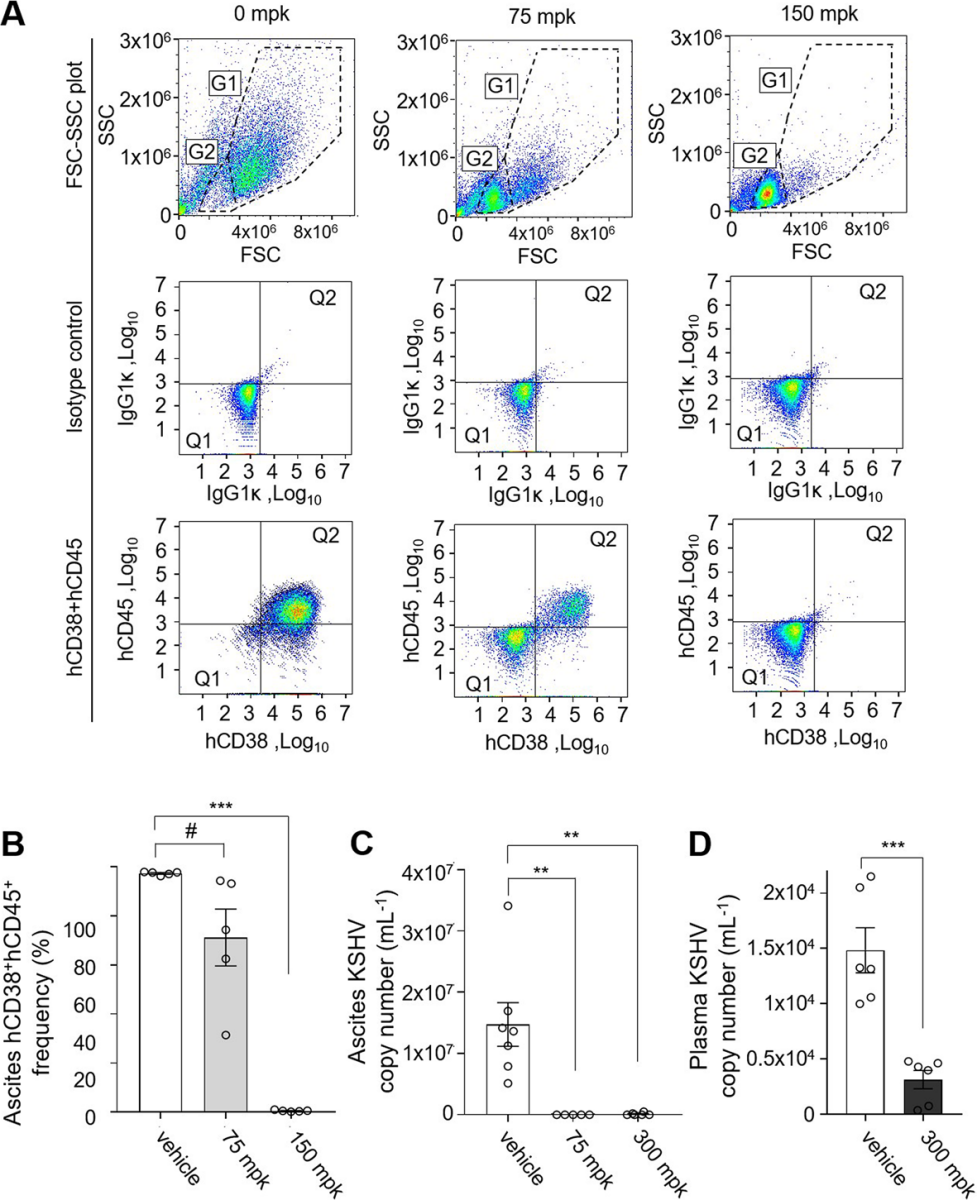
The Suppression of the ascites BCBL-1 proliferation and the KSHV propagation in vivo by the FIT-039 treatment. **A**-**B** Representative images (**A**) and the frequency plot (**B**) for the ascites from the PEL xenograft mice at day 43. Human CD38-PE and CD45-APC positive cells are counted as the BCBL-1-derived cells. n = 5 for each group. **C-D** The KSHV copy number was quantified by the quantitative PCR for PEL chylous ascites (**C**) and plasma (**D**) from the BCBL-1 xenograft mice with the FIT-039 treatment. *n* = 6, *n* = 5, and *n* = 6 for vehicle, 75 mpk, and 300 mpk for ascites, and *n* = 6 each for plasma at the day 45. Mean ± SE are shown; **, *p* < 0.01; ***, *p* < 0.001 by one-way ANOVA with Turkey’s multiple comparisons test; #, *p* < 0.05 by one-tailed Student’s t-test in (**B**). Mean ± SE are shown; **, *p* < 0.01; ***, *p* < 0.001 by two-tailed Student’s t-test in (**C**) and (**D**). mpk for mg/kg-BW in (**A**-**D**)

Finally, we conducted cell frequency analysis by FCM for chylous ascites from mice treated with 0, 75, and 150 mg/kg-BW FIT-039. We confirmed the dose-dependent reduction of intraperitoneal cell frequency of BCBL-1 cells, which were double positive for human CD38 and CD45 [33] (G1 of FSC-SSC plot and Q2 for double positive of human CD38 and CD45 in Fig. 4A), from 97% (0 mg/kg-BW) to 71% (75 mg/kg-BW) and 0.6% (150 mg/kg-BW), accompanied by increase in frequency of host mouse cells (G2 of FSC-SSC plot and Q1 for human CD45 and CD38-negative population in Fig. 4A) (Fig. 4A and B). The above observations confirmed that in the PEL xenograft model, resembling the microenvironment for PEL proliferation, FIT-039 administration exhibited a therapeutic effect.

### Suppression of the KSHV viral load in the PEL xenograft mice following the FIT-039 administration

We next examined whether FIT-039 administration resulted in the suppression of KSHV viral loads in PEL xenograft model mice. We evaluated the KSHV viral copy number in the chylous ascites and plasma from the peripheral blood of BCBL-1-xenografted mice treated with FIT-039 or vehicle. We observed suppression of the KSHV copy number by more than 99% in the chylous ascites of mice treated with 75 or 300 mpk FIT-039 (Fig. 4C), and by 79% in the plasma from 300 mpk FIT-039-treated mice (Fig. 4D), over the vehicle control group, confirming that the CDK9 inhibition by the FIT-039 results in suppression of the KSHV viral load in the BCBL-1 xenograft PEL, concordant with the anti-KSHV effects observed in vitro (Fig. 2A).

## Discussion

Previous studies have indicated that KSHV viral proteins function in the initiation of tumorigenesis [1, 2, 9]; however, their roles in subsequent proliferation remain unclear. In this study, we demonstrated that the CDK9 inhibitor FIT-039 suppressed the transcription of these KSHV viral genes, and administration of FIT-039 to the KSHV^+^ PEL xenograft model of BCBL-1 cells led to drastic inhibition of proliferation. We have previously demonstrated that CDK9 is required for the replication of HHV-1 (24). The observations described in this manuscript indicate that CDK9 is deeply involved in the proliferation of KSHV. With the absence of obvious somatic driver mutations in whole genome sequencing data for BCBL-1 cells, our observations suggest the importance of viral gene expression for PEL proliferation. In KSHV-associated malignancies, cells in the lytic phase play a major role in tumorigenesis [7]. While the functions of KSHV viral genes in cancer cell proliferation do not remain fully elucidated, a recent concept of a paracrine carcinogenesis model may provide a reasonable explanation [28]. In this model, growth-promoting paracrine factors released from the lytic cells facilitate the proliferation of surrounding cancer cells [28–32]. Concordant with this, clinical studies have shown that KSHV^+^ cancer patients benefit from the inhibition of vascular endothelial growth factor (VEGF) and platelet-derived growth factor (PDGF) paracrine signaling primed by lytic cells with imatinib and rapamycin treatment [34–36]. FIT-039 may suppress the expression of KSHV viral genes that play essential roles in these paracrine signals. It is also possible that kinases other than CDK9 are potentially involved in the antiviral effect of FIT-039 in a synergistic manner, as discussed previously [25]. The clinical benefits of inhibiting viral replication with nucleoside analogs vary according to reports and are not conclusive for PEL [37–41]. Since nucleoside analogs do not prevent the expression of viral genes, their limited or failed response may be due to viral genes and paracrine factors that are not targeted by nucleoside analogs. In this regard, a therapeutic benefit can be expected for FIT-039, which inhibits viral gene expression during the transcription step.

Moreover, the wide antiviral spectrum of FIT-039 provides clinical benefits for targeting KSHV-related cancers. As demonstrated in previous studies, FIT-039 also exhibits antiviral effects against human immunodeficiency virus type 1 (HIV-1) [22], in which viral propagation is highly dependent on P-TEFb [42–44]. HIV-1 infection facilitates the development of KSHV-associated oncogenesis, not only by inducing immune deficiency but also by inducing inflammatory cytokine responses through HIV-1 Tat [45–48]. Therefore, the control of HIV-1 viremia by highly active antiretroviral therapy (HAART), which is currently the standard therapy for AIDS-associated KSHV^+^ cancer [49–51] could be augmented by co-treatment with FIT-039. Furthermore, it is also known that Tat facilitates reactivation of the KSHV lytic replication cycle [52–56], and reciprocal activation of the propagation between HIV-1 and KSHV has also been reported [57–60]. Therefore, simultaneous targeting of multiple viruses with FIT-039 may provide therapeutic benefits.

To target wide-ranging virus-related symptoms, we developed FIT-039 as a skin patch, cervical tablet, and systemic administration drug, and FIT-039 is currently undergoing phase I/II trials for human papillomavirus-associated viral warts by skin patch [26, 27] and for cervical intraepithelial neoplasia with the cervical tablet, in Japan and South Korea [61]. Based on the anti-cancer activity of FIT-039 for KSHV^+^ malignancies ascertained in this study, the clinical trial of FIT-039 is currently being prepared for KSHV^+^ PEL and MCD with the FIT-039 intraperitoneal infusion, and KS with skin patch or systemic administration. However, in order to consider clinical applications, a careful assessment will be needed to determine an appropriate administration schedule of FIT-039 for KSHV suppression, since current xenograft study cannot fully reproduce the time-course of PEL or KS progression.

Therefore, the current study provides a rationale for the clinical application of FIT-039 for KSHV-associated malignancies to fulfil the unmet clinical needs.

## Supplementary Information

**Additional file 1:** **Supplementary Fig. S1.** Full images of RT-PCR. Full gel images are indicated for BCBL-1 in Fig. 1B. **Supplementary Fig. S2.** Full images of RT-PCR. Full images are indicated for BC-3 in Fig. 1B. **Supplementary Fig. S3.** Full images of RT-PCR and western blots. Full images are indicated for RT-PCR data in Fig. 1E and G, and western blots in Fig. 1I. **Supplementary Fig. S4.** Representative histochemical data for peritoneum, liver, and spleen for BCBL-1 xenografted mice and age-matched control mouse. HE staining and immunohistochemistry for human GAPDH are shown. Bars indicate 1 mm for light- field and 100 µm for fluorescent images.

**Additional file 2:** **Supplementary Table S1.** The list of primers used in this study.

**Additional file 3:** **Supplementary Table S2.** Incidence rates of potential metastasis in peritoneum.

## Data Availability

The whole-genome sequencing data of BCBL-1 and BC-3 cells obtained in this study is available through the Sequence Read Archive (SRA) of the National Center for Biotechnology Information (NCBI) BioProject with accession number PRJNA814425 (https://www.ncbi.nlm.nih.gov/bioproject/PRJNA814425) and PRJNA907516 (https://www.ncbi.nlm.nih.gov/bioproject/PRJNA907516), respectively. The other data and materials generated in the present study may be requested to the corresponding author through a material transfer agreement.

## Abbreviations

BRAF: B-Raf proto-oncogene serine/threonine kinase
CDK9: Cyclin-dependent kinase 9
CHEK2: Checkpoint kinase 2
CHOP: Cyclophosphamide, doxorubicin, vincristine, and prednisone
D-PBS: Dulbecco’s phosphate-buffered saline
ELK: ETS like-1
EPOCH: Etoposide, prednisone, vincristine, cyclophosphamide, and doxorubicin
HAART: Highly active antiretroviral therapy
HIV-1: Human immunodeficiency virus-1
kg-BW: Mg/kg body weight
KS: Kaposi’s sarcoma
KSHV: Kaposi’s sarcoma-associated herpes virus
MCD: Multicentric Castleman’s disease
PEL: Primary effusion lymphoma
P-TEFb: Positive transcription elongation factor
TPA: 12-O-tetradecanoylphorbol-13-acetate
