# Supplementary material for: Application of the CDK9 inhibitor FIT-039 for the treatment of KSHV-associated malignancy
Source: BMC Cancer. 2023 Jan 20;23:71. doi: 10.1186/s12885-023-10540-y (PMC9862866; doi:10.1186/s12885-023-10540-y)
Supplement: Supplementary file 1 — Additional file 1: Supplementary Fig. S1. Full images of RT-PCR. Full gel images are indicated for BCBL-1 in Fig. 1B. Supplementary Fig. S2. Full images of RT-PCR. Full images are indicated for BC-3 in Fig. 1B. Supplementary Fig. S3. Full images of RT-PCR and western blots. Full images are indicated for RT-PCR data in Fig. 1E and G, and western blots in Fig. 1I. Supplementary Fig. S4. Representative histochemical data for peritoneum, liver, and spleen for BCBL-1 xenografted mice and age-matched control mouse. HE staining and immunohistochemistry for human GAPDH are shown. Bars indicate 1 mm for light- field and 100 µm for fluorescent images. [file 12885_2023_10540_MOESM1_ESM.pdf]

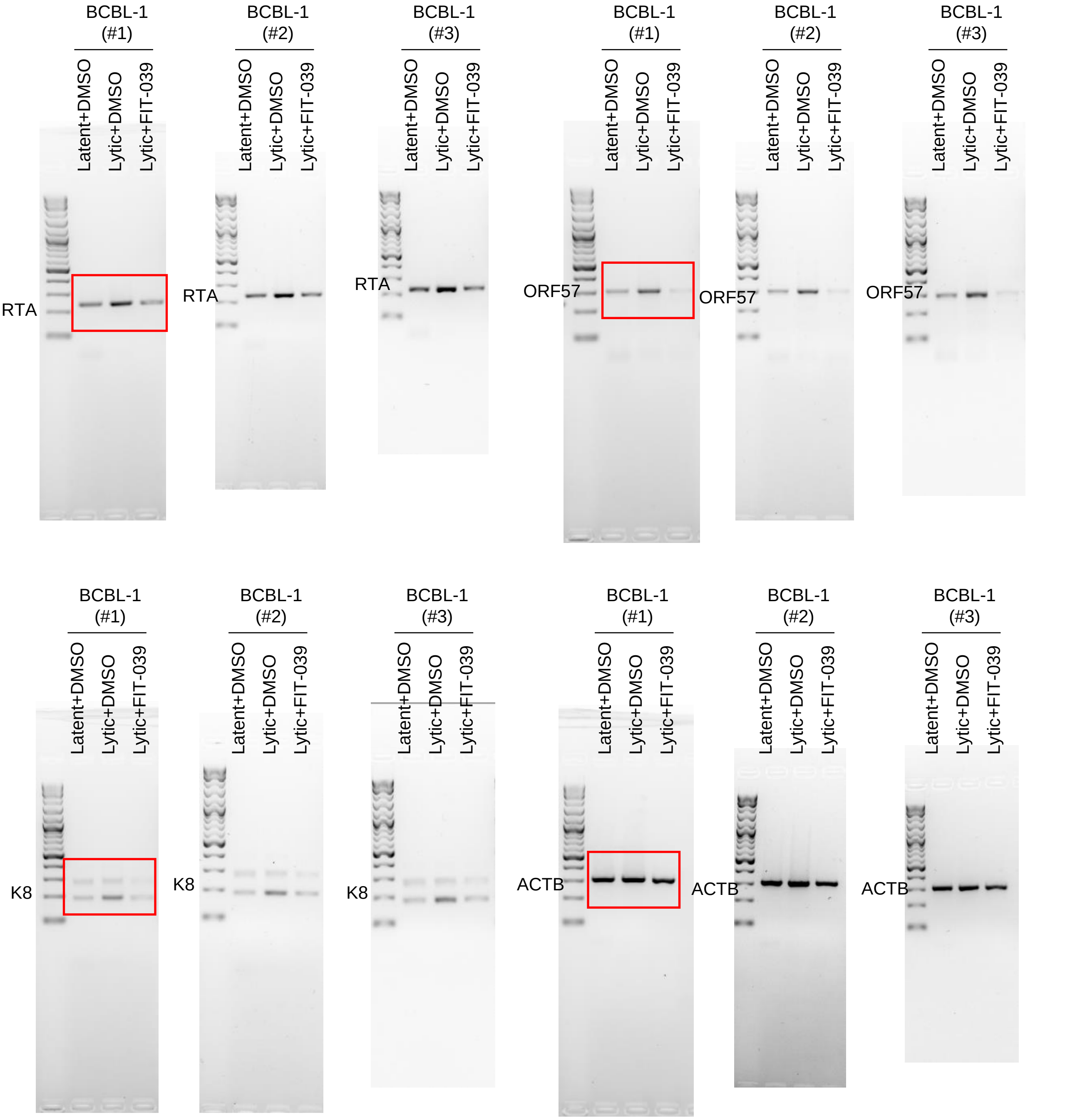

Supplementary Fig. S1. Full images of RT-PCR. Full gel images are indicated for BCBL-1 in Fig. 1B.

Source data: Fig. 1B, BC-3

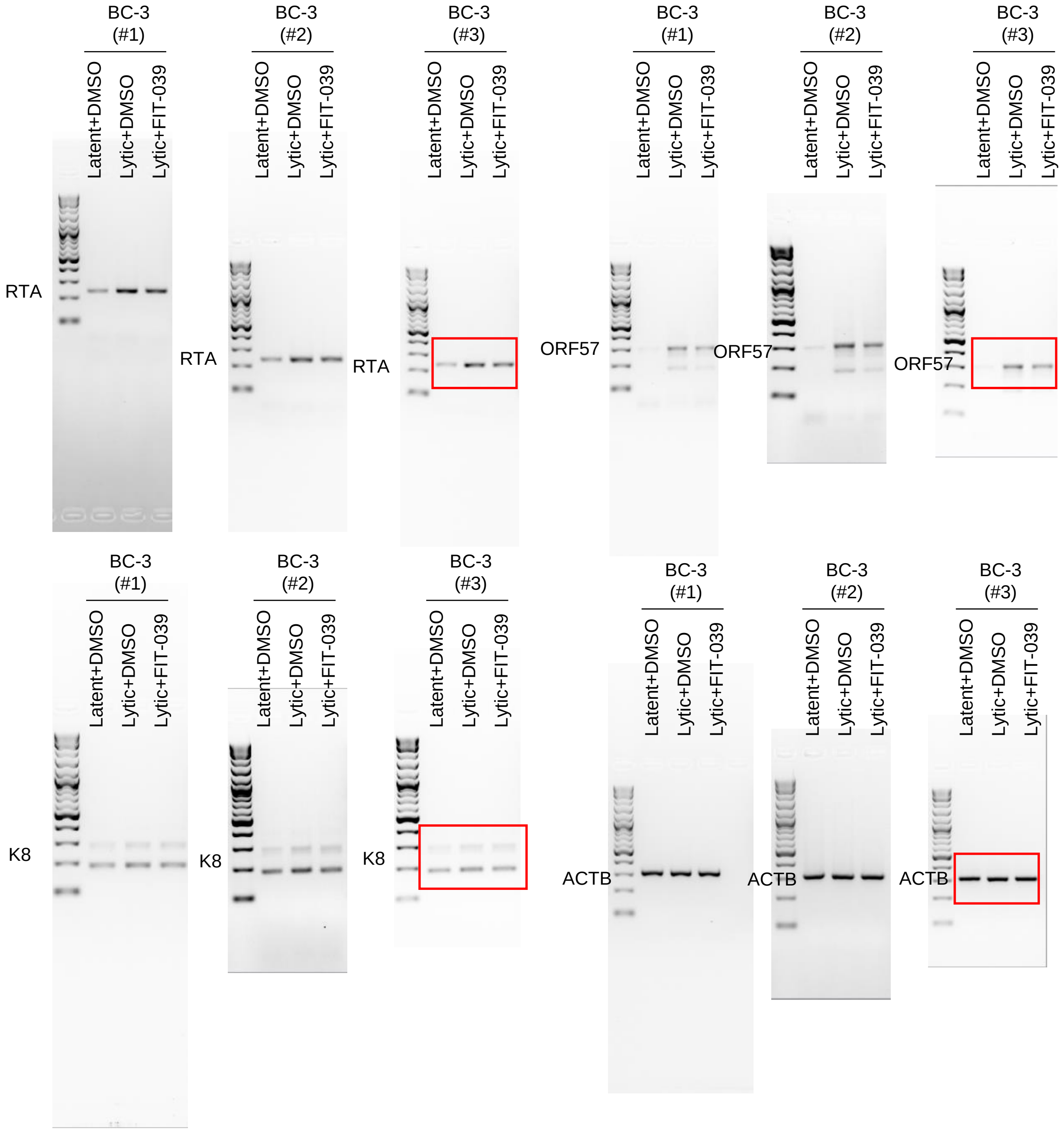

Supplementary Fig. S2. Full images of RT-PCR. Full images are indicated for BC-3 in Fig. 1B.

Source data: Fig. 1E

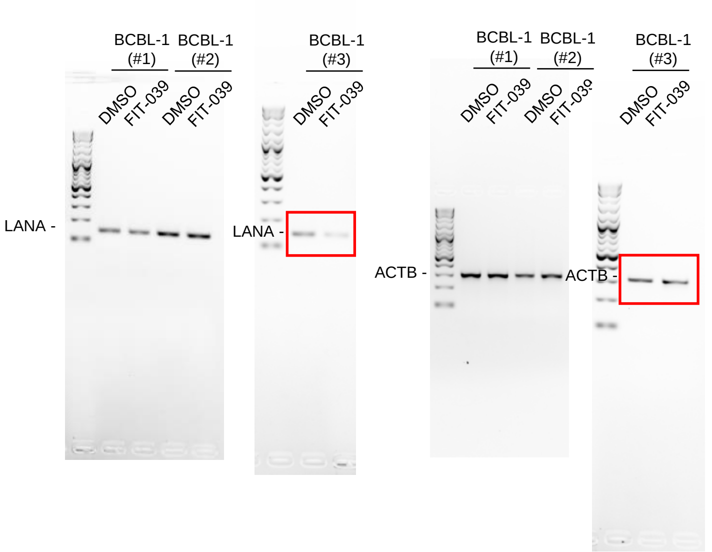

Source data: Fig. 1G

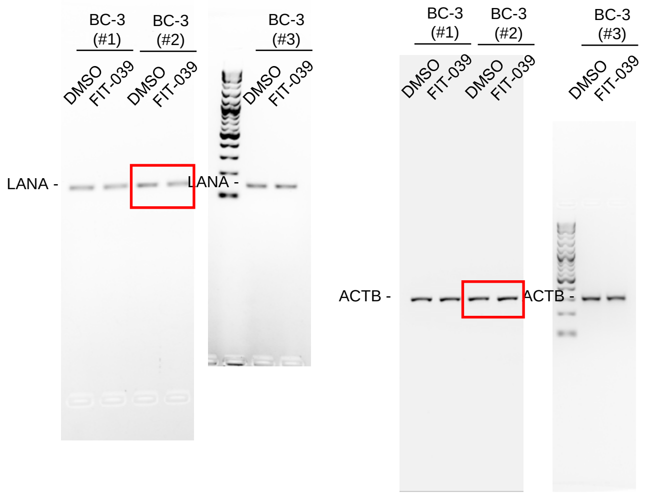

Source data: Fig. 1I

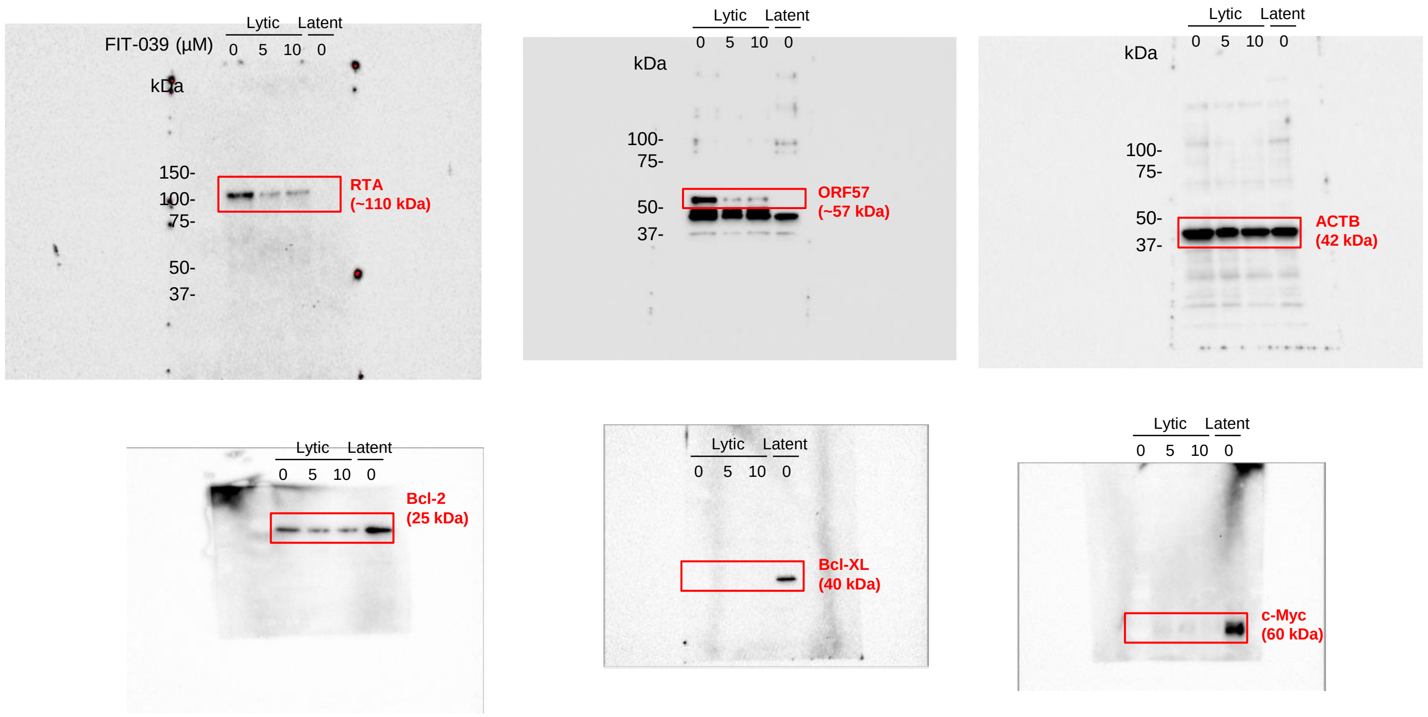

Supplementary Fig. S3. Full images of RT-PCR and western blots. Full images are indicated for RT-PCR data in Fig. 1E and 1G, and western blots in Fig. 1I.

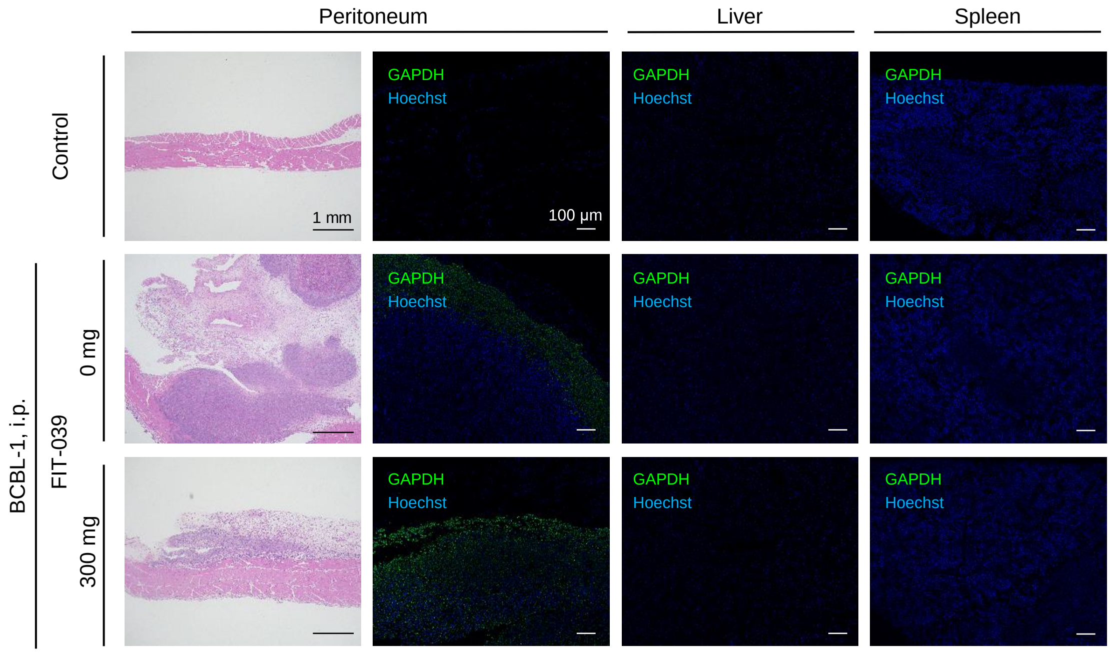

Supplementary Fig. S4. Representative histochemical data for peritoneum, liver, and spleen for BCBL-1 xenografted mice and age-matched control mouse. HE staining and immunohistochemistry for human GAPDH are shown. Bars indicate 1 mm for light-field and 100 μm for fluorescent images.
