# Supplementary material for: Application of the CDK9 inhibitor FIT-039 for the treatment of KSHV-associated malignancy
Source: BMC Cancer. 2023 Jan 20;23:71. doi: 10.1186/s12885-023-10540-y (PMC9862866; doi:10.1186/s12885-023-10540-y)
Supplement: Supplementary file 2 — Additional file 2: Supplementary Table S1. The list of primers used in this study. [file 12885_2023_10540_MOESM2_ESM.docx]

| Supplementary Table S1. The list of primers used in this study. | | | |  |
| --- | --- | --- | --- | --- |
| Target | Primer name | Direction | Sequence (5' to 3') | Used for |
| ACTB (H. Sapiens) | oAM13 | Forward | CCAACCGCGAGAAGATGACC | RT-PCR |
| ACTB (H. Sapiens) | oAM14 | Reverse | AGCTTCTCCTTAATGTCACG | RT-PCR |
| K-bZIP/K8 | oAM162 | Forward | TATGTGATCAGTCACATTCT | RT-PCR |
| K-bZIP/K8 | oAM163 | Reverse | TGGCACATTCGCATCAGCAT | RT-PCR |
| ORF57 | oAM99 | Forward | CGCACCGACACTGGAAGACG | RT-PCR |
| ORF57 | oAM100 | Reverse | ATTGTAGGCGGTCGCGTGGT | RT-PCR |
| *RTA* | oAM101 | Forward | CCAACTCTACCAGTGTGTGC | RT-PCR, real-time PCR |
| *RTA* | oAM102 | Reverse | CCTCTGCGCATGGCACGTTG | RT-PCR |
| *RTA* | oAM159 | Forward | (FAM)-GACGCATACGAAACAATCTACGATCCCAGTGA-(TAMRA) | real-time PCR (probe) |
| *RTA* | oAM158 | Reverse | CATAATCCGAATGCACACAT | real-time PCR |
