# Supplementary material for: Application of the CDK9 inhibitor FIT-039 for the treatment of KSHV-associated malignancy
Source: BMC Cancer. 2023 Jan 20;23:71. doi: 10.1186/s12885-023-10540-y (PMC9862866; doi:10.1186/s12885-023-10540-y)
Supplement: Supplementary file 3 — Additional file 3: Supplementary Table S2. Incidence rates of potential metastasis in peritoneum. [file 12885_2023_10540_MOESM3_ESM.docx]

Supplementary Table S2. Incidence rates of potential metastasis in peritoneum.

| Inoculation | FIT-039 | Incidence |
| --- | --- | --- |
| No | 0 mg/kg-BW | 0/10 (0%) |
| No | 300 mg/kg-BW | 0/10 (0%) |
| BCBL-1 | 0 mg/kg-BW | 9/10 (90%) |
| BCBL-1 | 300 mg/kg-BW | 6/10 (60%) |
